# Exploration of Novel Markers in Tan Sheep Spermatogenesis

**DOI:** 10.3390/ani16020350

**Published:** 2026-01-22

**Authors:** Yuan Ma, Haoyan Jin, Nana Wang, Yaru Xie, Lingkai Zhang, Bei Cai

**Affiliations:** 1 College of Animal Science and Technology, Ningxia University, Yinchuan 750021, China; myuan1809@163.com (Y.M.); m15939801218_1@163.com (H.J.); w2044590818@163.com (N.W.); xieyaru0724@163.com (Y.X.); 2 Key Laboratory of Ruminant Molecular Cell Breeding, Yinchuan 750021, China

**Keywords:** spermatogonia, spermatocytes, spermatozoa, markers, Tan sheep

## Abstract

In livestock farming, the reproductive function and breeding performance of Tan sheep are crucial for enhancing farming efficiency. Despite advances in research on sheep germ cells, studies on the identification of markers for spermatogonia, spermatocytes, and spermatozoa in Tan sheep remain limited and inadequate. In this study, Tan sheep were used as research subjects to investigate the morphological characteristics of testicular tissues, the developmental status of germ cells, and potential novel markers for spermatogonia, spermatocytes, and spermatozoa across different ages (0 days, 60 days, 180 days, and 365 days). Homology of the *SMC*3, *G*3*BP*1, and *AKAP*4 genes was analyzed via NCBI alignment. The localization and expression characteristics of these genes in the testis tissues of Tan sheep were investigated using HE staining, qPCR, and immunofluorescence double staining. The results showed that from 0 to 365 days of age, with increasing age, spermatogonia, spermatocytes, and spermatids exhibited an orderly distribution, and mature spermatozoa appeared in the tubular lumen, marking the initial establishment of the spermatogenic process. The homology of *SMC*3, *G*3*BP*1, and *AKAP*4 was 90%, 85%, and 81%. The mRNA levels of *SMC*3 and *G*3*BP*1 in the testes of 60-day-old Tan sheep were significantly increased, while *AKAP*4 expression showed a gradual increase with advancing age. SMC3 was co-localized with PLZF in undifferentiated spermatogonia, G3BP1 was co-expressed with SYCP2 in spermatocytes, and AKAP4 was co-expressed with PNA in spermatozoa. The findings of this study provide further supportive evidence for novel markers of spermatogonia, spermatocytes, and spermatozoa in Tan sheep.

## Simple Summary

During the reproductive process of Tan sheep, the normal development and functional realization of spermatogonia, spermatocytes, and spermatozoa are crucial for ensuring reproductive efficiency. In-depth research on the regulatory mechanisms underlying the development of Tan sheep germ cells represent a vital basis for improving reproductive performance. However, current studies on these germ cells are hampered by a paucity of available markers for identification and investigation, along with multiple limitations associated with the existing ones. The present study characterized the developmental dynamics of germ cells across different ages in Tan sheep and explored novel markers specific to ovine spermatogonia, spermatocytes, and spermatozoa.

## 1. Introduction

Tan sheep have emerged as one of the most important sheep breeds in China for meat and wool production, and they are primarily distributed in northern China. With its tender meat, high-quality fur, and strong adaptability, the Tan sheep possesses irreplaceable genetic value for the conservation of livestock genetic diversity and the development of characteristic industries [1,2,3]. After years of directional breeding and research, relevant technical achievements have been gradually applied in breeding practices, providing technical support for optimizing its production performance parameters. However, the breed’s inherent late-maturing trait has not been fundamentally improved, and its reproduction-related indicators remain suboptimal, specifically manifested as follows: the age of sexual maturity is approximately 6–8 months; the age at first mating is mostly delayed to over 12 months; the average semen concentration of rams is 2.5 × 10^9^/mL with sperm motility only ranging from 60% to 70%; and the annual lambing rate of ewes is less than 110%, accompanied by low body weight and slow weight gain [4,5]. These breed-specific reproductive shortcomings severely restrict the improvement of reproductive efficiency and have become the core bottleneck limiting the full exploitation of its genetic potential. Therefore, systematic investigation into the regulatory mechanisms underlying the reproductive function and performance of Tan sheep holds substantial theoretical significance and practical value for advancing breed improvement and increasing breeding efficiency.

Spermatogenesis constitutes the core process underlying male reproductive function. The spermatogenesis cycle in a male sheep takes 48 days. This process initiates in spermatogonia, among which the spermatogonia with stemness maintain the homeostasis of the spermatogonial stem cell pool, while the remaining subset differentiates into spermatocytes. These primary spermatocytes then enter meiosis to generate haploid round spermatids, which eventually develop into mature spermatozoa that are released into the lumen of the seminiferous tubules [6,7,8]. The orderly transition and functional homeostasis of germ cells at each stage are critical guarantees for the normal production of spermatozoa. In-depth analysis of the biological characteristics and developmental rules of these germ cells can provide core theoretical support for the precise improvement of reproductive performance, and exert universal guiding significance for enhancing the reproductive efficiency of breeding stock in animal husbandry.

At present, research on the identification and developmental mechanisms of sheep germ cells remains hampered by a lack of specific markers; this bottleneck is particularly pronounced in studies related to Tan sheep. Commonly used markers include THY1 and PLZF for spermatogonia; γH2AX and SYCP2/3 for spermatocytes; PRM1/PRM2 and PNA for spermatozoa; as well as the universal germ cell marker DDX4 (also known as VASA) [9,10,11]. Their limitations are mainly manifested as follows: first, some markers are under-expressed in other cell types, which impairs the accuracy of cell sorting and identification; second, they cannot effectively distinguish between different stages of spermatocyte meiosis, making it difficult to support studies on developmental dynamics; and third, they are susceptible to factors such as sperm maturity and preservation conditions, resulting in poor reproducibility of experimental results. Therefore, the identification of germ cell markers in Tan sheep serves as a critical prerequisite for breaking through current research bottlenecks and further elucidating its spermatogenic mechanisms.

Previous studies have identified that SMC3, G3BP1, and AKAP4 are specifically highly expressed in sheep spermatogonia, spermatocytes, and spermatozoa [12,13]. However, whether these molecules exhibit distinct stage-specificity during spermatogenesis in Tan sheep and can serve as reliable novel cellular markers remains to be elucidated. This study aims to explore and validate the feasibility of SMC3, G3BP1, and AKAP4 as markers for male germ cells in Tan sheep through interspecific gene homology analysis. Specifically, we first compared the conservation of mouse *SMC*3, *G*3*BP*1, and *AKAP*4 genes with their homologous sequences in Tan sheep using the NCBI database. Subsequently, qPCR was employed to detect the expression levels of these three genes in Tan sheep testicular tissues, and immunofluorescent double staining was used to clarify their cellular localization characteristics. This study is expected to provide novel candidate markers for the accurate identification of Tan sheep spermatogonia, spermatocytes, and spermatozoa, thereby offering theoretical basis and technical support for elucidating the molecular mechanisms of spermatogenesis in Tan sheep and breaking through the existing bottlenecks in germ cell identification technology.

## 2. Materials and Methods

### 2.1. Collection of Testicular Samples

In this study, male Tan sheep at 0, 60, 180, and 365 days of age were selected. The sheep were purchased from Tianyuan Agriculture and Animal Husbandry Technology Development Co., Ltd., located in Hongsibu District, Wuzhong City, Ningxia Hui Autonomous Region, China. The collected testes were rinsed with phosphate-buffered saline (PBS). Fresh testicular tissues were harvested and stored in dry ice for subsequent qPCR assays; the remaining tissues were transported to the laboratory in phosphate-buffered saline supplemented with double the amount of penicillin/streptomycin. Testicular tissue samples (1 cm^3^) were fixed in Bouin’s solution for subsequent paraffin embedding experiments. All experimental animals and procedures involved in this study have been approved by the Science and Technology Ethics Committee of Ningxia University.

### 2.2. Histological Examination

Testicular tissues fixed in Bouin’s solution for approximately 16 h were subjected to embedding. The tissues were dewaxed with xylene, dehydrated using ethanol with increasing concentrations, and cleared with xylene. After being immersed in wax at 68 °C, the tissues were embedded. Following embedding, tissue sections were cut consecutively at a thickness of 7 μm using the Leica RM2245 microtome (Wetzlar, Germany) and mounted on adhesive slides. Sections were rehydrated through a series of ethanol solutions with decreasing concentrations. After rehydration, the sections were stained using a hematoxylin–eosin staining kit (G1120, Beijing, China) and then dehydrated with ethanol solutions of increasing concentrations followed by xylene. Finally, the sections were mounted with neutral balsam (G8590, Beijing, China). Images were observed and captured using an OLYMPUS BX43 microscope (Tokyo, Japan) with cellSens Entry software (version 4.4). All experiments were repeated three times.

### 2.3. Immunofluorescence Staining

Use a microtome to continuously cut tissue sections 5 μm thick and mount them onto adhesive slides for immunofluorescence staining. Non-identical species sources: After undergoing paraffin removal with xylene, ethanol rehydration, EDTA repair, and BSA blocking, samples were incubated overnight at 4 °C with appropriate primary antibodies (Table 1). Following incubation, secondary antibodies were applied to visualize immunofluorescence. Secondary antibodies used in this experiment: Alexa Fluor 594 AffiniPure Goat Anti-Mouse IgG (H+L) (1:200, WA0307011), Alexa Fluor 488 AffiniPure Goat Anti-Rabbit IgG (H+L) (1:200, WA0307031), Dylight 488 Goat Anti-Mouse IgG (H+L) (1:200, A23210), and Dylight 594 Goat Anti-Rabbit IgG (H+L) (1:200, A23420). TSA Fluorescence Double Staining (RK05902, Wuhan, China). Homologous Species Source: Follow the same steps as before primary antibody incubation. After labeling the secondary antibody with HRP from the TSA Fluorescent Double-Staining Kit (RK0590, Wuhan, China), add fluorescein and incubate to visualize immunofluorescence. After first-round antibody staining, antibodies were washed to eliminate cross-reactions. Following thorough washing, second-round antibody incubation and staining were performed. Finally, nuclei were stained with DAPI, and slides were mounted with an antifade mounting medium. Images were observed and captured using an OLYMPUS BX43 microscope with cellSens Entry software. All antibody suppliers are from China, and all experiments were replicated three times. All experiments were repeated three times.

### 2.4. Quantitative Real-Time Polymerase Chain Reaction

Fresh frozen testis tissues of Tan sheep at different postnatal ages were ground thoroughly. Total RNA was extracted using the Trizol reagent method [30,31]. Reverse transcription was performed with the PrimeScript™ FAST RT reagent Kit with gDNA Eraser (Cat. No. RR092A, Takara Bio Inc., Dalian, China) to convert total RNA into complementary DNA (cDNA), which effectively eliminated genomic DNA contamination. qPCR was carried out using the SYBR Green dye method, with β-actin serving as the reference gene (housekeeping gene) to normalize the expression levels of target genes. Data processing and statistical analysis were performed using GraphPad Prism software (Version 9.5.0). Differences in the relative expression levels of target genes among groups of different ages were analyzed by one-way analysis of variance (one-way ANOVA). The specific statistical method was as follows: The relative expression level of the target gene was calculated using the 2^−ΔΔCt^ method (Livak method), where ΔCt = Ct value of the target gene − Ct value of the reference gene (*β*-*actin*), and ΔΔCt = ΔCt value of the experimental group − ΔCt value of the control group. This method was used to normalize the expression level of the target gene to that of the reference gene. A value of *p* < 0.05 was considered statistically significant, and *p* < 0.01 was considered extremely statistically significant. All primers used in this study were synthesized by Sangon Biotech (Shanghai) Co., Ltd., China (Table 2). All experiments were repeated three times.

## 3. Results

### 3.1. Histological Analysis of Testicular Tissue in Tan Sheep at Different Ages

**Figure 1:**
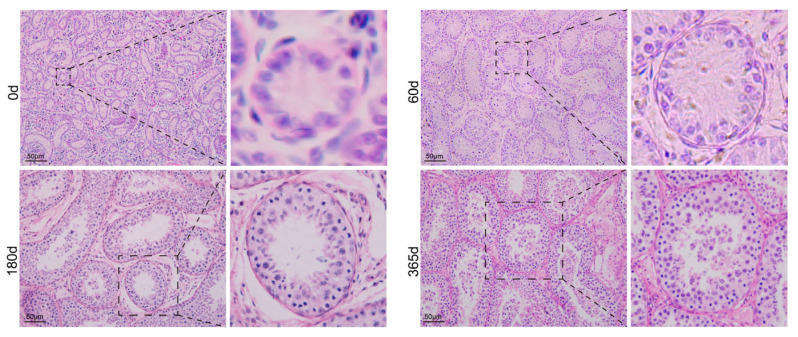
Developmental status of testis tissue in Tan sheep at different ages. Scale bar = 50 μm.

To investigate the developmental status of testis tissue in Tan sheep at different ages, testicular tissues from Tan sheep at 0, 60, 180, and 365 days after birth (i.e., 4 developmental stages: Neonatal stage, Juvenile stage, Sexual maturity stage, Somatic maturity stage) were examined, followed by histological analysis (Figure 1). From 0 to 365 days of age, the diameter of the seminiferous tubules in Tan sheep testes showed a tendency to expand. At 0 days of age, the testicular tissue structure was not yet fully developed; the seminiferous tubules only formed tiny luminal structures, and no differentiation was observed. During the period from 60 to 180 days of age, the lumens of the seminiferous tubules expanded significantly with clear structures. Proliferation of spermatogonia was observed, and spermatocytes appeared. Meanwhile, the number of Leydig cells increased, their cytoplasm showed a pink color after staining, and Sertoli cells gradually developed into a mature state. From 180 to 365 days of age, spermatogonia, spermatocytes, and spermatids were observed to be distributed in an orderly manner, and mature sperm appeared in the lumens, marking the initial establishment of the spermatogenesis process.

### 3.2. Development of Spermatogonia in Testes of Tan Sheep at Different Ages

**Figure 2:**
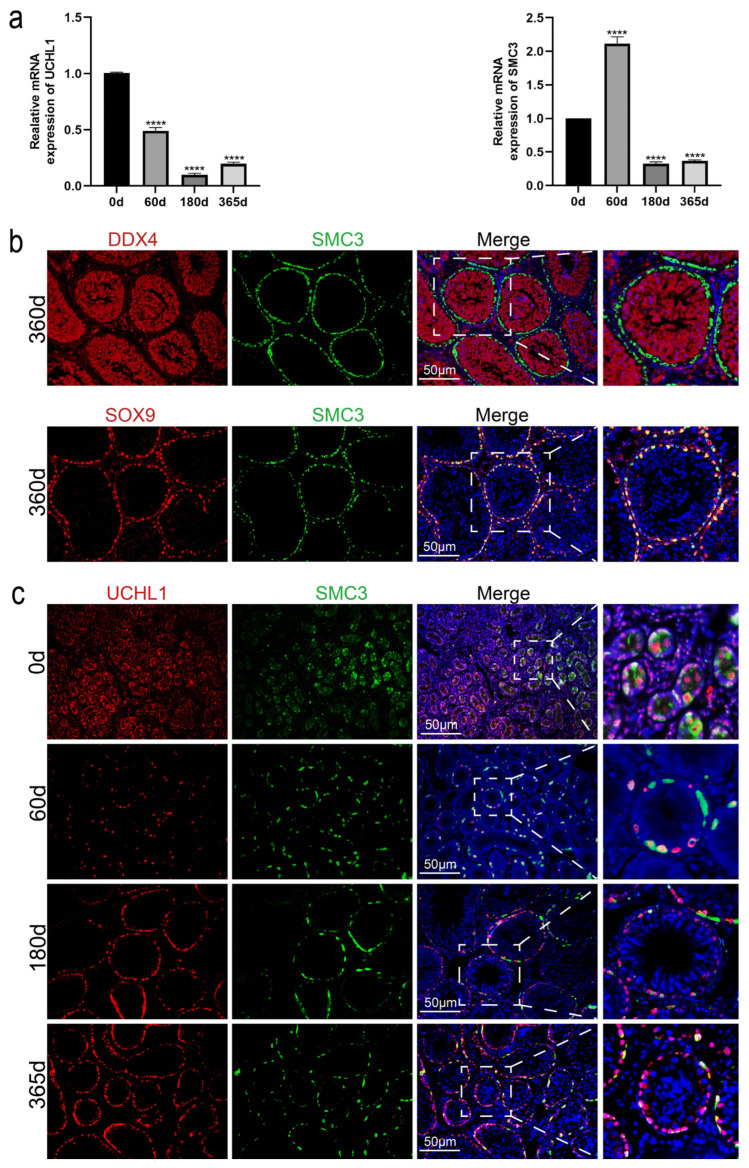

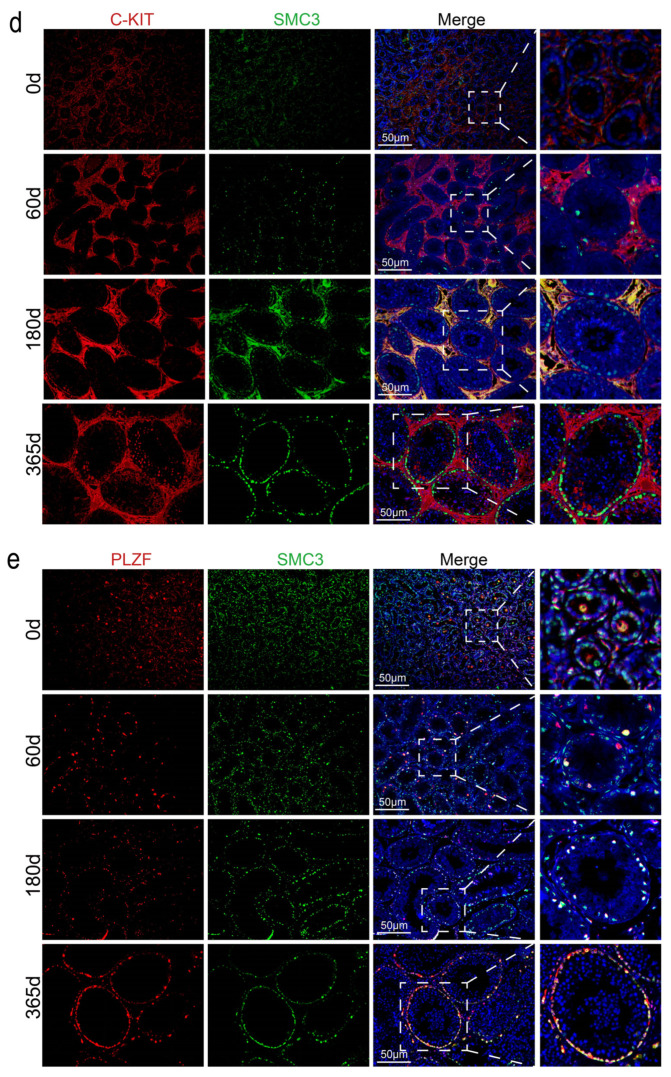
Development of spermatogonia in testes of Tan sheep at different ages. (**a**) Expression of *UCHL*1 and *SMC*3 in testis tissue of Tan sheep at different ages. Data are presented as mean ± standard error of the mean (SEM), n = 3. Statistical analysis was performed using one-way ANOVA followed by Dunnett’s post hoc test for multiple comparisons against the 0 d control group. Significance levels: ** *p* < 0.01, *** *p* < 0.001, **** *p* < 0.0001 vs. 0 d group. (**b**) DDX4/SMC3 and SOX9/SMC3 immunofluorescence co-staining of testis tissue from Tan sheep at 365 days old. (**c**) UCHL1/SMC3 immunofluorescence co-staining of testis tissue from Tan sheep at different ages. (**d**) C-KIT/SMC3 immunofluorescence co-staining of testis tissue from Tan sheep at different ages. (**e**) PLZF/SMC3 immunofluorescence co-staining of testis tissue from Tan sheep at different ages. Nuclei were stained with DAPI (blue). Scale bar = 50 μm.

Mouse *SMC*3 shares 90% homology with that of the Tan sheep. Relative mRNA expression results of *UCHL*1 (a spermatogonial marker) and *SMC*3 in testicular tissues of Tan sheep at different ages show that, when normalized to the 0-day baseline, the expression level of *UCHL*1 decreased significantly while that of *SMC*3 increased significantly at 60 days of age; at 180 days of age, both *UCHL*1 and *SMC*3 expression levels decreased significantly; at 365 days of age, the increase in *UCHL*1 and *SMC*3 expression levels was not significant (Figure 2a).

Subsequently, we further explored the expression characteristics of SMC3 in testicular tissue. Although male sheep at sexual maturity can produce sperm, they exhibit low sperm density and weak motility, with spermatogenesis being initially established but not yet mature. However, the sperm production and quality of sheep stabilize after 365 days of age. Therefore, testicular tissue from 365-day-old Tan sheep was selected for subsequent experiments. We performed DDX4/SMC3 and SOX9/SMC3 immunofluorescence co-staining assays on testicular tissue from 365-day-old Tan sheep (Figure 2b). The results showed that DDX4 was co-expressed with SMC3, and SOX9 was partially co-expressed with SMC3, indicating that SMC3 is highly expressed in both spermatogonia and Sertoli cells.

Based on this result, to further determine whether SMC3 can serve as a marker for spermatogonia in Tan sheep, we investigated the localization and expression profiles of SMC3 in Tan sheep testicular tissue. Accordingly, we performed UCHL1/SMC3 immunofluorescence co-staining assays on testicular tissues of Tan sheep at different ages (Figure 2c). The results demonstrated that from 60 to 365 days of age, UCHL1 and SMC3 exhibited co-expression in spermatogonia. To more precisely clarify whether SMC3 is localized in differentiated or undifferentiated spermatogonia, we further conducted C-KIT/SMC3 and PLZF/SMC3 immunofluorescence co-staining assays (Figure 2d,e). The results revealed that from 60 to 365 days of age, co-expression of PLZF and SMC3 occurred, which clearly confirmed that SMC3 is highly expressed in undifferentiated spermatogonia. SMC3 may synergistically regulate the self-renewal of spermatogonial stem cells with PLZF.

### 3.3. Development of Spermatocytes in Testes of Tan Sheep at Different Ages

**Figure 3:**
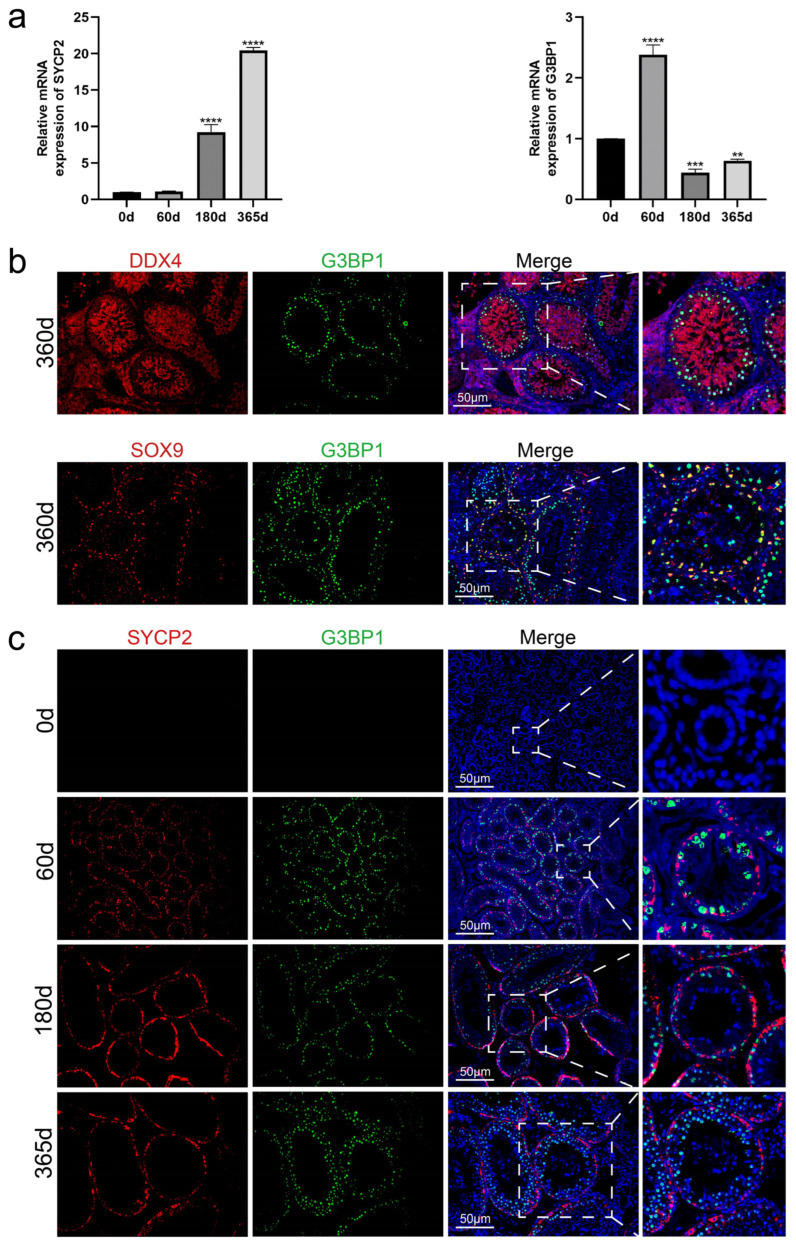
Development of spermatocytes in testes of Tan sheep at different ages. (**a**) Expression of *SYCP*2 and *G*3*BP*1 in testis tissue of Tan sheep at different ages. Data are presented as mean ± standard error of the mean (SEM), n = 3. Statistical analysis was performed using one-way ANOVA followed by Dunnett’s post hoc test for multiple comparisons against the 0 d control group. Significance levels: ** *p* < 0.01, *** *p* < 0.001, **** *p* < 0.0001 vs. 0 d group. (**b**) DDX4/G3BP1 and SOX9/G3BP1 immunofluorescence co-staining of testis tissue from Tan sheep at different ages. (**c**) SYCP2/G3BP1 immunofluorescence co-staining of testis tissue from Tan sheep at different ages. Nuclei were stained with DAPI (blue). Scale bar = 50 μm.

Mouse *G*3*BP*1 shares 85% homology with that of the Tan sheep. Relative mRNA expression results of *SYCP*2 (a spermatocyte marker) and *G*3*BP*1 in testicular tissues of Tan sheep at different ages show that, with the expression level at 0 days as the baseline for normalization, the expression level of *G*3*BP*1 increased significantly at 60 days of age; at 180 days of age, the expression level of *SYCP*2 increased significantly, while the expression level of *G*3*BP*1 was extremely low; at 365 days of age, the expression level of *SYCP*2 further increased to the peak, and the expression level of *G*3*BP*1 rose (Figure 3a).

Subsequently, we further investigated the expression characteristics of G3BP1 in testicular tissues. We performed immunofluorescence co-staining assays for DDX4/G3BP1 and SOX9/G3BP1 on testicular tissues of 365-day-old Tan sheep (Figure 3b). The results showed that DDX4 was co-expressed with G3BP1, while SOX9 and G3BP1 exhibited partial co-localization.

Based on this result, to further determine whether G3BP1 can serve as a specific marker for spermatocytes in Tan sheep, we explored the localization and expression characteristics of G3BP1 in Tan sheep testicular tissues. Therefore, we conducted SYCP2/G3BP1 immunofluorescence co-staining assays on testicular tissues of Tan sheep of different ages (Figure 3c). The results indicated that with the increase in age, from 60 days to 180 days of age, G3BP1 and SYCP2 showed co-expression; notably, at 365 days of age, the expression of G3BP1 toward the lumen increased significantly, preliminarily identifying that G3BP1 is highly expressed in spermatocytes.

### 3.4. Development of Spermatozoa in Testes of Tan Sheep at Different Ages

**Figure 4:**
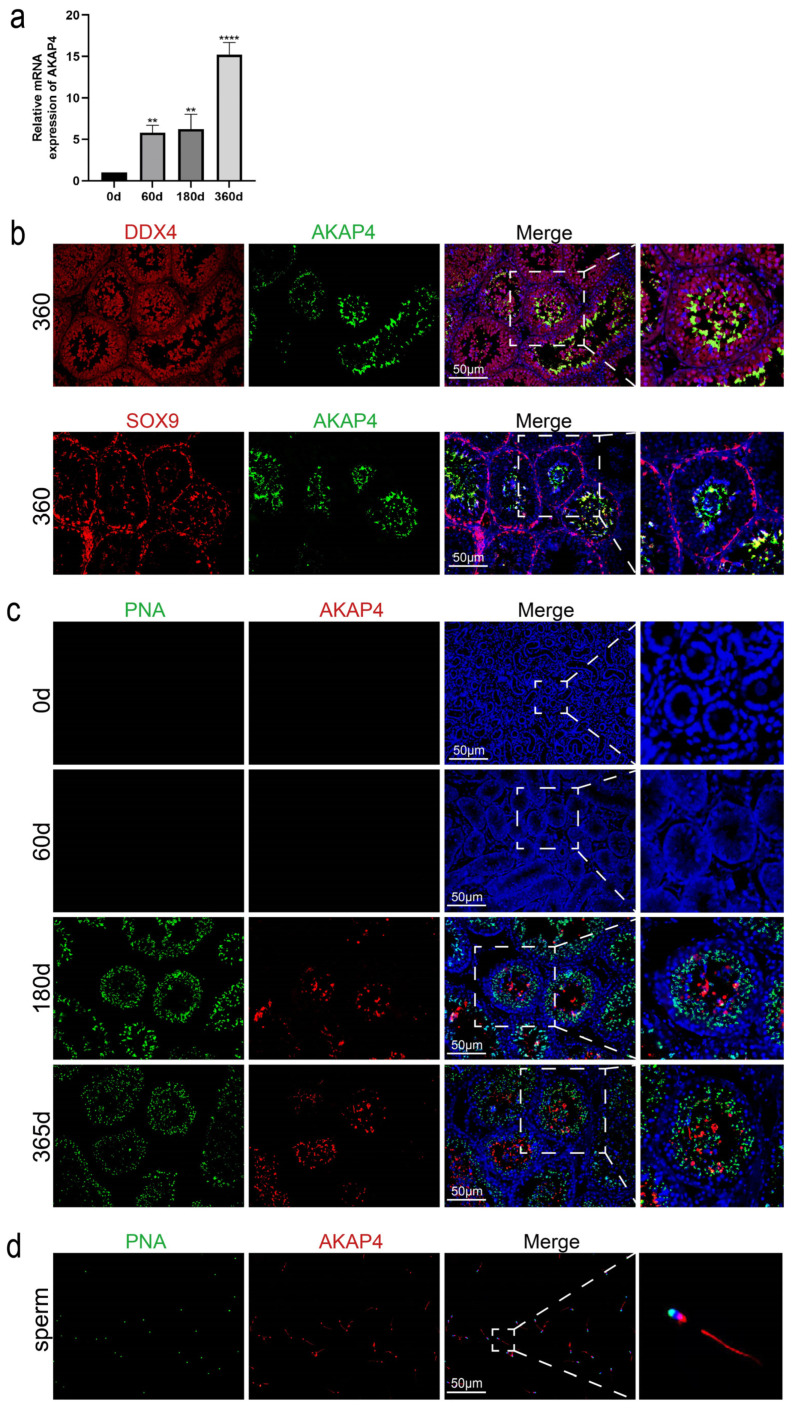
Development of sperm in testes of Tan sheep at different ages. (**a**) Expression of *AKAP*4 in testis tissue of Tan sheep at different ages. Data are presented as mean ± standard error of the mean (SEM), n = 3. Statistical analysis was performed using one-way ANOVA followed by Dunnett’s post hoc test for multiple comparisons against the 0 d control group. Significance levels: ** *p* < 0.01, *** *p* < 0.001, **** *p* < 0.0001 vs. 0 d group. (**b**) DDX4/AKAP4 and SOX9/AKAP4 immunofluorescence co-staining of testes from Tan sheep. (**c**) PNA/AKAP4 immunofluorescence co-staining of testis tissue from Tan sheep at different ages. (**d**) AKAP4 immunofluorescence staining of mature sperm from Tan sheep. Nuclei were stained with DAPI (blue). Scale bar = 50 μm.

Mouse *AKAP*4 shares 81% homology with that of the Tan sheep. Relative mRNA expression results of *AKAP*4 in testicular tissues of Tan sheep at different ages show that, with the expression level at 0 days as the baseline for normalization, the expression level of *AKAP*4 was extremely low at 60 days of age; at 180 days of age, the expression level increased significantly; and at 365 days of age, the expression level further increased to the peak value (Figure 4a).

Subsequently, we further explored the expression characteristics of AKAP4 in testicular tissues. We performed immunofluorescence co-staining assays for DDX4/AKAP4 and SOX9/AKAP4 on testicular tissues of 365-day-old Tan sheep (Figure 4b). The results showed that AKAP4 is localized in germ cells and has no co-expression with SOX9.

Based on this result, to further determine whether AKAP4 can serve as a specific marker for spermatids in Tan sheep, we investigated the localization and expression characteristics of AKAP4 in Tan sheep testicular tissues. Therefore, we conducted PNA/AKAP4 immunofluorescence co-staining assays on testicular tissues of Tan sheep of different ages (Figure 4c). The results indicated that with increasing age, AKAP4 and PNA showed co-expression during the period from 180 to 365 days of age. However, the specific location of AKAP4 expression in Tan sheep sperm remained unclear. Thus, we performed immunofluorescence staining on Tan sheep sperm, and the results revealed that it is expressed in the sperm flagellum (Figure 4d).

## 4. Discussion

Spermatogenesis in mammals is a complex and dynamic process of cell differentiation [32]. In studies related to sheep, specific markers for spermatogonia, spermatocytes, and spermatozoa have been widely reported. However, research on cell markers associated with spermatogenesis in Tan sheep, a specific breed, remains relatively scarce [33,34,35]. Against this backdrop, the functional characterization of several core markers has been established in this field. PLZF acts as a key transcription factor in undifferentiated spermatogonia and represents a canonical marker for the maintenance of stem cell self-renewal [19]. c-KIT specifically labels differentiated spermatogonia and participates in cell differentiation signaling pathways [18]. UCHL1 is a key molecule in the identification and differentiation regulation of spermatogonia, maintaining the balance of spermatogonial differentiation and spermatogenesis [20]. SYCP2 is a core component of the synaptonemal complex in spermatocytes, signifying the progression of meiosis [21,22]. Meanwhile, FITC-PNA serves as a marker for the acrosome of spermatozoa [23,24]. Existing studies have demonstrated that SMC3, G3BP1, and AKAP4 are all closely associated with male reproductive function. SMC3 is a core subunit of the cohesin complex, mediating the segregation of sister chromatids during mitosis and meiosis; depletion of SMC3 in mice leads to embryonic developmental failure and an infertile phenotype [25,26]. As a stress granule assembly factor, G3BP1 is involved in DNA binding and cyclic GMP-AMP synthase activation in early spermatocytes of mice, and its functional abnormalities may interfere with meiotic progression [27]. AKAP4 is an essential molecule for the structure and function of the sperm fibrous sheath; deletion of AKAP4 in mice results in abnormal sperm morphology and motility, accompanied by infertility [28,29].

**Figure 5:**
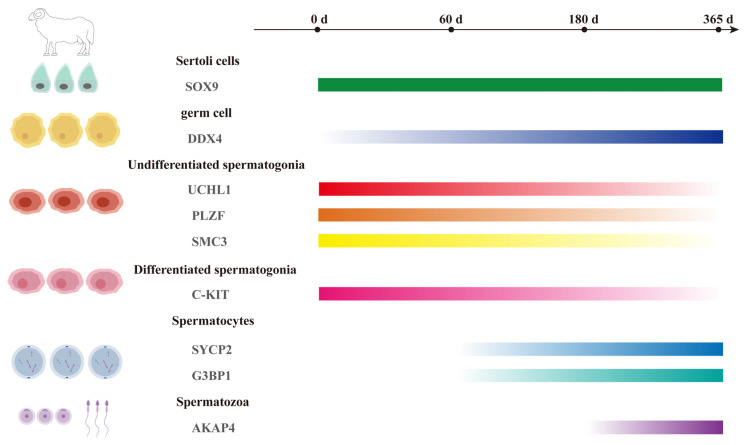
A schematic overview illustrating the primary findings of this study.

In this study, we investigated the morphological characteristics of Tan sheep testicular tissues, the developmental status of germ cells, and potential novel markers for spermatogonia, spermatocytes, and spermatozoa across different ages (0 days, 60 days, 180 days, and 365 days). Studies have shown that both SOX9 and DDX4 are expressed during the reproductive process in mice. Among these, SOX9 is closely associated with the function of Sertoli cells in the mouse testis, and influences the structure and function of seminiferous tubules [36]. Additionally, the deletion of SOX9 in mouse testes leads to XY sex reversal (male-to-female) [37,38]. DDX4 is involved in the regulation of germ cell differentiation and development [39]. Our findings are consistent with the aforementioned studies: SOX9 and DDX4 are constitutively expressed in the testes of Tan sheep, which implies they may exert similar roles in the Tan sheep testis. Additionally, we detected the expression of UCHL1, PLZF, and SMC3 in undifferentiated spermatogonia. In the mouse testis, knockout of MAST4 leads to a decrease in PLZF expression, which in turn impairs the cell cycle progression of spermatogonial stem cells [40]. This observation highlights the critical role of PLZF in the development of mouse germ cells. Currently, research on SMC3 has focused on tissues such as the hematopoietic system and cardiac development [41,42], while its role in germ cells requires further investigation. C-KIT is expressed in differentiated spermatogonia and persists until later stages of spermatogenesis [43]. SYCP2 and G3BP1 are expressed in spermatocytes [44,45,46], and AKAP4 is expressed in spermatozoa [47]. These results are consistent with previous studies conducted in mice (Figure 5). The results of this study provide further supporting evidence for the new markers of spermatogonia, spermatocytes, and spermatozoa in Tan sheep, and also lay an important theoretical foundation for the optimization of reproductive technologies and genetic improvement in the livestock sector.

### 4.1. Spermatogonia

The mRNA level of *SMC*3 significantly increased in 60-day-old Tan sheep testes. This elevation may be attributed to the protein’s role within the cohesin complex, which serves as a key regulator of proper chromosome segregation. An elevated level of this complex can ensure the stable transmission of chromosomes in rapidly dividing cells, thereby preventing cell death or mutations caused by abnormal chromosome segregation [26,48].

Our study found that SMC3 is expressed in both germ cells and Sertoli cells of Tan sheep, revealing that SMC3 may be involved in the interaction of spermatogenic epithelial cells, and this distribution pattern is consistent with the role of SMC family proteins in maintaining chromatin structure [41]. Further co-localization analysis using the spermatogonial marker UCHL1 revealed partial co-localization between SMC3 and UCHL1. However, the expression of SMC3 exhibited more prominent changes during specific developmental stages (e.g., postnatal days 0–60). Notably, UCHL1 and SMC3 differed in their expression timing and cellular specificity, which may be attributed to their distinct roles in spermatogenesis. The co-localization of PLZF and SMC3 may imply a cooperative interaction between these two proteins in specific biological processes of spermatogonia. It is hypothesized that during SSC self-renewal, PLZF may either recruit SMC3 or act synergistically with SMC3 on specific chromatin regions to modulate chromatin structure and function. This modulation further regulates the expression of target genes, thereby maintaining the biological characteristics of SSCs. In future studies, the specific molecular mechanisms underlying the interactions of PLZF and C-KIT with SMC3, respectively, should be further investigated. Additionally, exploring the conservation and divergence of these interactions across different species will provide a more robust theoretical basis for research in the field of reproductive science.

### 4.2. Spermatocytes

The mRNA level of *G*3*BP*1 in the testes of 60-day-old Tan sheep increased significantly, which might be attributed to physiological stresses (e.g., oxidative stress, endoplasmic reticulum stress) induced by rapid germ cell proliferation, elevated metabolic rate, and changes in the hormonal microenvironment during this developmental stage [49].

We observed that G3BP1 is expressed in both germ cells and Sertoli cells of Tan Sheep. As an RNA-binding protein (RBP), the expression of G3BP1 in Sertoli cells suggests that it may be involved in the post-transcriptional regulation of SOX9 target gene mRNAs. Partial co-localization was observed between SYCP2 and G3BP1, which may be associated with the role of SYCP2 as a marker for meiotic initiation and progression. Combined with existing studies, it is hypothesized that this co-localization is likely involved in the dynamic regulation of RNA during homologous chromosome synapsis. G3BP1 may participate in the SYCP2-mediated synaptonemal complex assembly process by regulating the translation efficiency of specific mRNAs [50,51]. In animal reproduction, dysregulation of this mechanism may lead to meiotic arrest, thereby impairing spermatogenesis. The expression of G3BP1 in primary spermatocytes of Tan Sheep serves as a core molecular basis for mitigating environmental stress and sustaining normal meiotic progression. This finding provides a novel molecular perspective for elucidating the differences in reproductive performance among sheep breeds. Currently, research on G3BP1 in sheep remains extremely limited. In subsequent studies, Tan Sheep-specific models (e.g., CRISPR-based gene editing) could be established to validate its functional role. Additionally, the development of high-sensitivity G3BP1 detection methods (such as flow cytometry combined with enzyme-linked immunosorbent assay, ELISA) is critical for enabling its clinical application.

### 4.3. Spermatozoa

The mRNA level of *AKAP*4 in the testes of Tan sheep exhibits an age-dependent increase. This phenomenon may be attributed to the gradual maturation of the spermatogenic epithelium in the testes and the elevated proportion of spermatids as age advances. Upon entering the spermiogenesis stage (e.g., acrosome formation and tail elongation), a large amount of AKAP4 needs to be synthesized to construct the fibrous sheath. This structure not only provides support and protection for the sperm tail but also anchors cyclic adenosine monophosphate (cAMP)-dependent protein kinase (PKA), thereby regulating phosphorylation signals associated with sperm motility. Consequently, the expression of AKAP4 increases synchronously with the maturation of spermatids [52,53].

PNA/AKAP4 exhibits a co-expression pattern, particularly in the 180–365 day age range, with both molecules localized to specific regions of spermatozoa. Fang et al. generated an AKAP4 knockout mouse line using the CRISPR-Cas9 system; their findings revealed that loss of AKAP4 leads to abnormalities in sperm morphology and motility, ultimately resulting in male infertility [28,29]. However, across multiple animal species including mice, sheep, cattle, horses, and dogs, the concentration of pro-AKAP4 (ProAKAP4) shows a significant positive correlation with sperm motion parameters such as total motility and curvilinear velocity (VCL). In sperm samples where ProAKAP4 concentration decreases after cryopreservation, sperm motility is also significantly reduced, and this suggests that ProAKAP4 could serve as a marker for cryotolerance of Tan sheep spermatozoa [54,55,56,57,58]. Furthermore, AKAP4 participates in signal transduction cascades through interactions with molecules such as cyclic adenosine monophosphate (cAMP)-dependent protein kinase (PKA). Given that the acrosome reaction involves a series of complex signal transduction processes, the glycoproteins bound by PNA may be associated with the signaling pathway mediated by AKAP4, and together they regulate the occurrence of the acrosome reaction [59]. The underlying mechanism of this PNA/AKAP4 co-expression phenomenon warrants further investigation. Preliminary identification indicates that AKAP4 could serve as a potential marker for Tan sheep spermatozoa. Future studies should further dissect its regulatory mechanisms to advance the precision of Tan sheep reproductive technologies. Additionally, AKAP4 holds promise as a core target for enhancing the economic benefits of the Tan sheep industry.

In conclusion, in this study, we performed morphological, histological, and immunofluorescence analyses on the testes of Tan sheep aged from 0 to 365 days. We systematically investigated the testicular development of Tan sheep, with a particular focus on the development of spermatogonia, spermatocytes, and spermatozoa (Figure 5). From the perspective of practical application in Tan sheep breeding, SMC3 can identify spermatogonial stem cells. By utilizing SMC3 labeling to screen for high-quality breeding stock (such as those with high productivity, disease resistance, and superior genetic backgrounds), SSCs can be selected to establish a cryopreserved stem cell bank. This method enables long-term preservation of genetic resources, and is particularly suitable for endangered species or rare livestock. G3BP1, as a spermatogonial marker, can assess the meiotic process of germ cells in testes, providing molecular evidence for early prediction of breeding potential in rams. AKAP4, as a sperm marker, exhibits expression levels closely correlated with sperm structural integrity and motility. During semen cryopreservation, it can optimize cryoprotectant formulations and freeze–thaw protocols, thereby enhancing post-thaw fertilization capacity and ultimately improving artificial insemination success rates. Collectively, the combined application of these three markers (SMC3, G3BP1 and AKAP4) spans the entire Tan sheep breeding pipeline, encompassing early genetic selection, semen quality assessment, and elite germplasm propagation. It also facilitates the precise implementation of embryo transfer technology and accelerates the propagation of superior breeds [60,61,62]. In conclusion, the germ cell-specific markers identified in this study hold substantial theoretical significance and broad application prospects for advancing reproductive biology research in Tan sheep and promoting the high-quality development of the animal husbandry industry.

## 5. Conclusions

This study is the first to investigate the development of germ cells across different age stages in Tan sheep, as well as identify novel markers for spermatogonia, spermatocytes, and spermatozoa in this breed. The findings revealed that SMC3, G3BP1, and AKAP4 exhibit significant expression in undifferentiated spermatogonia, spermatocytes, and spermatozoa of Tan sheep. However, this study has certain limitations. Firstly, the research was primarily based on in vitro tests using testicular tissues, and the specific roles of these markers in the in vivo context of Tan sheep require further validation. Secondly, the synergistic effects between these newly identified markers and other known markers for spermatogonia, spermatocytes, and spermatozoa remain unclear. Future studies could employ gene knockout animal models to clarify their functions in the reproductive process of Tan sheep. In conclusion, the results of this study provide further supportive evidence for the identification of novel markers for spermatogonia, spermatocytes, and spermatozoa in Tan sheep.

## Figures and Tables

**Table 1:** Information on primary antibodies and probes used for immunofluorescent staining.

Antibody Name	Host Species	Dilution Ratio	Supplier and Catalog Number	Combination Protocol	Reference
DDX4	Rabbit	1:200	ABclonal, A0357, Shanghai, China	Co-incubated with SMC3, G3BP1, and AKAP4, respectively	[14,15]
SOX9	Rabbit	1:200	ABclonal, A19710, Wuhan, China	Co-incubated with SMC3, G3BP1, and AKAP4, respectively	[16,17]
C-KIT	Rabbit	1:200	ABclonal, A0357, Wuhan, China	data Co-incubated with SMC3	[18]
PLZF	Rabbit	1:200	ABclonal, A21185, Wuhan, China	Co-incubated with SMC3	[19]
UCHL1	Mouse	1:200	Abcam, AB307736, Shanghai, China	Co-incubated with SMC3	[20]
SYCP2	Rabbit	1:200	ABclonal, A16098, Wuhan, China	Co-incubated with G3BP1	[21,22]
FITC-PNA	-	1:200	Thermo Fisher, L32460, Shanghai, China	Co-incubated with AKAP4	[23,24]
SMC3	Rabbit	1:200	ABclonal, A19591, Wuhan, China	Co-incubated with UCHL1	[25,26]
G3BP1	Mouse	1:200	Proteintech, 66486-1-Ig, Wuhan, China	Co-incubated with SYCP2	[27]
AKAP4	Rabbit	1:200	ABclonal, A14813, Wuhan, China	Co-incubated with FITC-PNA	[28,29]

**Table 2:** All primer sequences.

Target Gene Name	Primer Type	Primer Sequence (5′ to 3′)
*β*-*actin*	Forward	GGCATCCTGACCCTCAAGTA
	Reverse	GGGGTGTTGAAGGTCTCAAA
*UCHL*1	Forward	ATTCAGGCAGCCCACGAT
	Reverse	GGCATCCGTCCATCAAGT
*SMC*3	Forward	TCACTTCGGAGAGTTATCGG
	Reverse	GTGCTGTTGCCATCTGGTTA
*SYCP*2	Forward	AGAATGCCTCAAGATGCCAG
	Reverse	CTGACAAGCCAGTTCTCGTCT
*G*3*BP*1	Forward	GTACCAGCTTCACAGCCTCG
	Reverse	ACATTTATTCGCTGTTCTCGC
*AKAP*4	Forward	GTCTAAGACGGAGGGATC
	Reverse	TGGAAACCTAAGGCGTAC

## Data Availability

The original contributions presented in this study are included in the article. Further inquiries can be directed to the corresponding authors.

## Abbreviations

The following abbreviations are used in this manuscript:

HE	Hematoxylin and Eosin Staining
qPCR	Quantitative Polymerase Chain Reaction
NCBI	National Center for Biotechnology Information
DDX4	DEAD-box helicase 4
SOX9	Sex Determining Region Y-Box 9
PLZF	Promyelocytic leukemia zinc finger
C-KIT	c-KIT receptor tyrosine kinase
UCHL1	Ubiquitin C-terminal Hydrolase L1
SYCP2/3	Synaptonemal Complex Protein 2/3
FITC-PNA	Fluorescein Isothiocya-nate-Labeled Peptide Nucleic Acid
SMC3	structural maintenance of chromosomes 3
G3BP1	G3BP stress granule assembly factor 1
AKAP4	A-kit nase anchoring protein 4
THY1	Thy-1 cell surface antigen
γH2AX	Spermato gonia; phosphorylated histone H2AX
PRM1/2	Protamine 1/2
SSC	Spermatogonial Stem Cell
